# Molecular and biochemical characterization of a novel isoprene synthase from *Metrosideros polymorpha*

**DOI:** 10.1186/s12870-018-1315-4

**Published:** 2018-06-15

**Authors:** Soo-Jin Yeom, Moonjung Kim, Seong Keun Kim, Dae-Hee Lee, Kil Koang Kwon, Hyewon Lee, Haseong Kim, Dong-Myung Kim, Seung-Goo Lee

**Affiliations:** 1 Synthetic Biology and Bioengineering Research Center, KRIBB, Daejeon, 34141 Republic of Korea; 2 Department of Chemical Engineering and Applied Chemistry, Chungnam National University, Daejeon, 34113 Republic of Korea; 3 Department of Biosystems and Bioengineering, KRIBB School of Biotechnology, University of Science and Technology, Daejeon, 34113 Republic of Korea

**Keywords:** Isoprene synthase, DMAPP, *Escherichia coli*, Mevalonate pathway

## Abstract

**Background:**

Isoprene is a five-carbon chemical that is an important starting material for the synthesis of rubber, elastomers, and medicines. Although many plants produce huge amounts of isoprene, it is very difficult to obtain isoprene directly from plants because of its high volatility and increasing environmental regulations. Over the last decade, microorganisms have emerged as a promising alternative host for efficient and sustainable bioisoprene production. Isoprene synthase (IspS) has received much attention for the conversion of isoprene from dimethylallyl diphosphate (DMAPP). Herein, we isolated a highly expressible novel IspS gene from *Metrosideros polymorpha* (MpIspS), which was cloned and expressed in *Escherichia coli*, using a plant cDNA library and characterized its molecular and biochemical properties.

**Results:**

The signal sequence deleted MpIspS was cloned and expressed in *E. coli* as a 65-kDa monomer. The maximal activity of the purified MpIspS was observed at pH 6.0 and 55 °C in the presence of 5 mM Mn^2+^. The *K*_m_, *k*_cat_, and *k*_cat_/*K*_m_ for DMAPP as a substrate were 8.11 mM, 21 min^− 1^, and 2.59 mM^− 1^ min^− 1^, respectively. MpIspS was expressed along with the exogenous mevalonate pathway to produce isoprene in *E. coli*. The engineered cells produced isoprene concentrations of up to 23.3 mg/L using glycerol as the main carbon source.

**Conclusion:**

MpIspS was expressed in large amounts in *E. coli*, which led to increased enzymatic activity and resulted in isoprene production in vivo. These results demonstrate a new IspS enzyme that is useful as a key biocatalyst for bioisoprene production in engineered microbes.

**Electronic supplementary material:**

The online version of this article (10.1186/s12870-018-1315-4) contains supplementary material, which is available to authorized users.

## Background

Isoprene (2-methyl-1,3-butadiene) is a volatile five-carbon terpene that is an important platform chemical in the synthetic chemistry industry for the synthesis of rubber, pharmaceuticals, flavors, and potential biofuels [1–5]. Almost all isoprene has been produced from petrochemical sources, mainly by direct isolation from C5 cracking fractions or through the dehydrogenation of C5 isoalkanes and isoalkenes [6, 7]. However, the C5 supply has been dependent upon the petroleum industry and the chemical production process is relatively energy-intensive and environment-unfriendly, and the yields may be insufficient for future demands [8].

To overcome these disadvantages, bioisoprene has become an attractive alternative. Bioisoprene is synthesized by isoprene synthase (IspS; EC 4.2.3.27) from dimethylallyl diphosphate (DMAPP). IspSs have been isolated and characterized from several plants, such as kudzu [9], poplars [10], aspen [11, 12], velvet bean [13], willows [5], and oaks [14]. Some IspSs have also been also employed to produce isoprene in *Escherichia coli* [15–19], *Saccharomyces cerevisiae* [20], *Synechocystis* [21], and *Bacillus subtilis* [22]. Although isoprene has been produced from these engineered microorganisms, their production level remains insufficient to satisfy the supply needed by industry [19]. As IspS is the key enzyme for the isoprene biosynthetic pathway needs to be established. Identification of new IspSs may provide enzymes with improved kinetics that will benefit the constructed microbial cell factories for efficient isoprene production.

In this study, our goal was to isolate a new IspS from a plant cDNA library and functionally characterize the IspS in *E. coli*. We mined transcriptome datasets of various plants to identify an IspS homologue. Among seven plants, a putative IspS was discovered from *Metrosideros polymorpha*, which emits volatile terpene [23]. However, emission of isoprene or IspS from *M. polymorpha* has not been reported. The gene encoding the putative IspS from *M. polymorpha* (MpIspS) was cloned and expressed in *E. coli*. The biochemical properties of the purified MpIspS, such as the effects of metal ions, pH, temperature, and kinetics, were investigated. Finally, the characterized MpIspS successfully produced isoprene in *E. coli* harboring an exogenous mevalonate (MVA) pathway. MpIspS could be used as a potential enzyme for the production of isoprene in an *E. coli* system.

## Results and discussion

### Discovery of new IspS

**Fig. 1:**
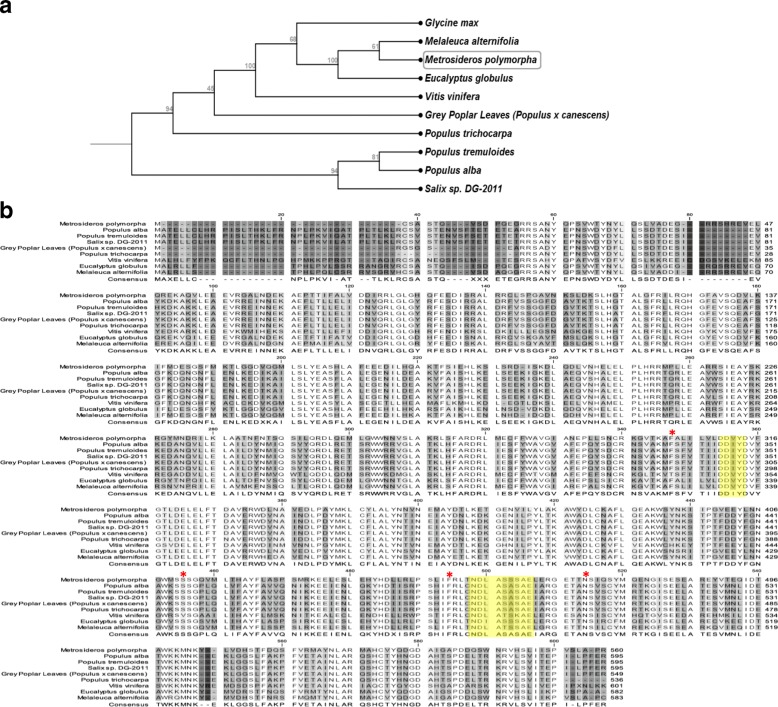
Phylogenetic tree and sequence alignment of the MpIspS with some known IspSs. **a** Phylogenetic tree of the retrieved MpIspS and known IspS sequences. The bars represent evolutionary distance. **b** Alignment of amino acid sequences of IspSs. The GenBank accession numbers are: *Populous alba*, ADG96473.1; *Populus tremuloides,* Q7XAS7.1; *Salix* sp., AEK70967.1; *Populus trichocarpa*, ACD70404.1; *Vitis vinifera,* CAN65805.1; *Eucalyptus globulus*, BAF02831.1; *Melaleuca alternifolia*, AAP40638.1. Shaded sequences are the metal ions binding motif, DDXXD, and NDXXTXXXE. The canonical amino acid residues for isoprene synthases are boxed

An IspS homologue was identified from a plant cDNA library using gene-mining (OmicsPia Co. Ltd., Daejeon, Korea). The detailed method for the mining of IspS is described in the Methods section. We identified an IspS from *M. polymorpha*, which is a species of flowering evergreen tree in the myrtle family. For the MpIspS in this study, sequence alignments were performed and analyzed in detail by comparison with previously reported IspS sequences. A phylogenetic tree and a multiple sequence alignment indicating key amino acid positions in various IspSs are shown in Fig. 1a and b. The other genes previously proposed as the *ispS* genes of *Eucalyptus grandis, Melaleuca alternifolia, Vitis vinifera, Camellia sinensis, Populus trichocarpa, Populus alba,* and *Populus x canadensis* exhibited 84, 86, 54, 54, 54, 54, and 54%, amino acid sequence identities, respectively (Fig. 1a). The multiple sequence alignment revealed the conservation of two metal ion-binding sites, an aspartate-rich DDXXD motif and an NDXXSXXXE (Fig. 1b), across all IspSs, including MpIspS. All known IspSs had an “isoprene score” as defined Sharkey et al. [24]. The devised score depends on how many of these four amino acids are the canonical F338, S445, F485, and N505. MpIspS had a score of 4, as shown by the star in Fig. 1b. In previous results, it has been suggested that these canonical residues are involved in substrate specificity [24, 25].

### Gene cloning, purification, and molecular mass determination

**Fig. 2:**
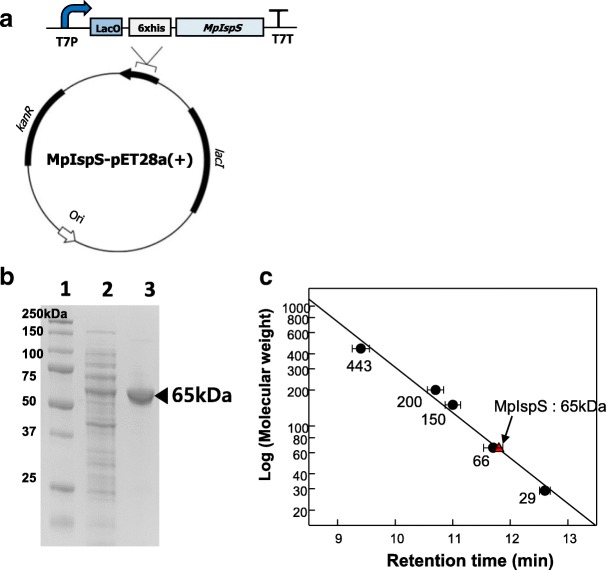
SDS-PAGE analysis and determination of the molecular mass of MpIspS. **a** Map of MpIspS. Essential components of the vector are indicated. The MpIspS coding region was cloned between the T7 promoter and terminator in vector pET-28a(+) containing the N-terminal His_6_ tag. **b** SDS-PAGE of MpIspS. Lanes: 1, pre-stained marker protein (250, 150, 100, 75, 50, 37, and 25 kDa); 2, crude extract; 3, purified enzyme. **c**. Determination of the molecular mass of MpIspS by gel-filtration chromatography. Reference proteins as closed circle were apoferritin (443 kDa), β-amylase (200 kDa), alcohol dehydrogenase (150 kDa), albumin (13.7 kDa), and carbonic anhydrase (29 kDa). MpIspS is represented by the red triangle

The gene encoding the MpIspS (1683 bp) was codon-optimized, synthesized, subcloned, and expressed in *E. coli* (Additional file 1: Figure S1 and Fig. 2a). The enzyme was purified as a soluble protein from the crude extract obtained from harvested cells by immobilized metal affinity chromatography (IMAC). The purified MpIspS enzyme exhibited a single band of approximately 65 kDa when analyzed by sodium dodecyl sulfate-polyacrylamide electrophoresis (SDS-PAGE) (Fig. 2b). This is consistent with the calculated molecular mass of 64,950 Da based on the 561 amino acid residues and six histidine residues as determined with the Compute pI/Mw tool [26]. The native molecular mass of the MpIspS determined using Superose 12 10/300 gel filtration chromatography was estimated to be 65 kDa as a monomer, based on the masses of reference proteins (Fig. 2c). In previous reports, the native molecular mass of IspSs from *P. tremuloides* and *P. alba* was reported as 121 kDa as a dimer [27] and 68 kDa as a monomer [10], respectively.

### Effects of metals on the activity of MpIspS

**Fig. 3:**
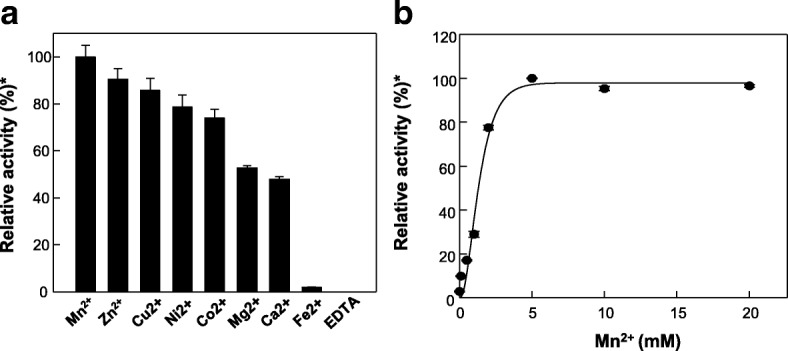
Effect of metal ions on the activity of MpIspS. **a** Effect of different metal ions on the activity of MpIspS. The reactions were performed in 50 mM MOPS buffer (pH 6.0) containing 50 μM DMAPP and 1 mM of each metal ion, at 50 °C for 10 min. **b** Effect of the concentration of Mn^2+^ on the activity of MpIspS. *Relative activity of 100% was 17.5271 U/mg

Various IspSs have been reported to require a divalent metal cofactor, such as a Mg^2+^ or Mn^2+^, for their activity, like other terpenoid synthases [10, 12]. Thus, we examined the effects that various divalent metals on MpIspS activity. The purified MpIspS enzyme displayed no activity following the removal of metal ions by treatment with ethylenediaminetetraacetic acid. Among the metal ions tested, Mn ^2+^ was the most effective for MpIspS activity, with an optimal concentration of 5 mM (Fig. 3a and b). By considering the highest activity with Mn^2+^ to be 100%, activities of 91, 86, 79, 74, 53, 48, and 2% were detected in the presence of Zn^2+^, Cu^2+^, Ni^2+^, Co^2+^, Mg^2+^, Ca^2+^, and Fe^2+^, respectively. The observation of maximal activity of MpIspS in the presence of Mn^2+^ was interesting, given that all IspSs that have been characterized prefer Mg^2+^ as a cofactor [10, 12, 13, 28]. In some cases, Mn^2+^ can partially substitute for Mg^2+^ [5, 12, 13]. The IspS from *Casuarina equisetifolia* [29]*, Campylopus introflexus* [30], and MpIspS (this study) displayed the highest activities in the presence of Mn^2+^, with the activities being less in the presence of Mg^2+^.

### Effects of pH and temperature on the activity of MpIspS

**Fig. 4:**
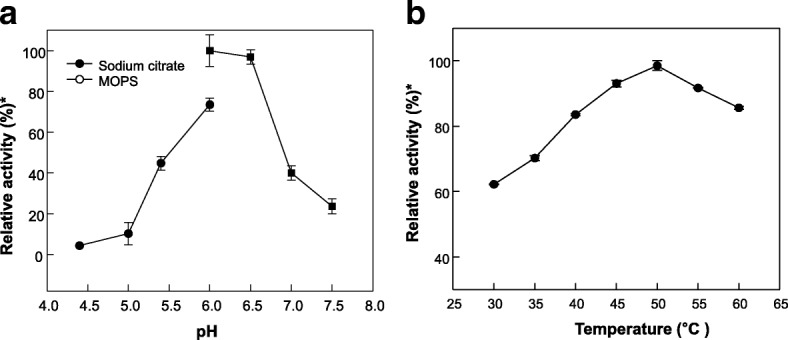
Effects of pH and temperature on the activity of MpIspS. **a** pH: The reactions were performed in 50 mM sodium citrate (*closed circle*) or 50 mM MOPS buffer (opened circle) containing 50 μM DMAPP and 5 mM Mn^2+^ at 55 °C for 10 min. **b** Temperature: Reactions were performed in 50 mM MOPS buffer (pH 6.0) containing 50 μM DMAPP and 5 mM Mn^2+^ of the enzyme for 10 min. *Relative activity of 100% was 17.5271 U/mg

The MpIspS activity was examined over a pH range of 4.5–7.5 containing 5 mM Mn^2+^ or 1 mM Mg^2+^. The maximal enzyme activity was observed at pH 6.0 and 5 mM Mn^2+^ (Fig. 4a). At pH 5.5 and 7.0, the activity was approximately 40% of the maximum. Additionally, we tested the pH dependency in the presence of Mg^2+^ favorable for IspS activity. Maximal enzyme activity was observed at pH 6.0–6.5 and 1 mM Mg^2+^ (Additional file 2: Figure S2). The effect of temperature on enzyme activity was investigated. Maximum activity was recorded at 50 °C (Fig. 4b). At temperatures of 30 and 45 °C, the activity was approximately 80% of the maximum. The maximum activity of various IspSs from *Populus alba* at pH 8 and 40 °C [10], *P. tremuloides* at pH 8 and 32 °C [11, 12], *Salix discolor* at pH 8 and 35 °C [31], *Campylopus introflexus* at pH 8.6 and 40 °C [30], *Casuarina equisetifolia* at pH 8 and 40 °C [29], *Ficus septica* at pH 9.5 and 40 °C [29], and *F. virgate* at pH 10 and 40 °C [29] have been reported. Interestingly, previously characterized IspSs have been demonstrated to be active at a broad range of pH values with pH optima between 7 and 10. In addition, IspSs appear to have temperature optimum in the 35–40 °C range. Monoterpene synthases display pH optima ranging from 6 to 7 [32–34], because monoterpene synthases activity was related to chloroplast pH in plants. An acidic chloroplast pH and temperature above 40 °C favors the emission of monoterpenes [35]. *M. polymorpha* generally grows in acidic, weathered soils where the pH may be as low as 3.6 [36] and in the presence of terpene emission [23]. These ecological may be related with the pH optimum of MpIspS for isoprene conversion.

### Kinetic parameters of MpIspS

**Fig. 5:**
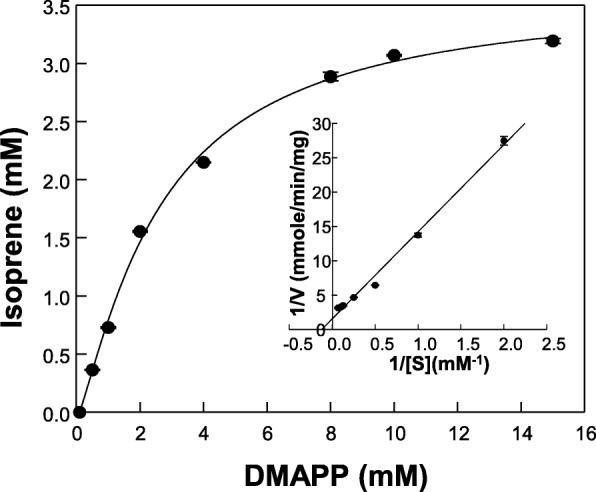
Michaelis–Menten kinetics Michaelis–Menten plot of MpIspS initial velocity versus substrate (DMAPP) concentration, monitoring production of isoprene. Reaction was in 50 mM MOPS buffer (pH 6.0) containing 5 mM Mn^2+^ and indicated concentration of DMAPP at 50 °C for 10 min

The kinetic parameters of purified recombinant MpIspS protein for DMAPP were determined. The curves of the Michaelis-Menten equation with the experimental data for DMAPP are shown in Fig. 5. MpIspS activity increased with increasing DMAPP concentration up to 15 mM, and higher concentrations inhibited the activity under our assay conditions. Therefore, the *K*_m_ value was estimated by non-linear curve fitting of Michaelis–Menten formula at concentrations below 15 mM. Consequently, the *K*_m_, *k*_cat_, and *k*_cat_/*K*_m_ of MpIspS for DMAPP as substrate were 8.11 mM, 21 min^− 1^, and 2.59 mM^− 1^ min^− 1^, respectively. Turnover rates (*k*_cat_) for other IspSs are 1.7 s^− 1^ for *P. tremuloides* [12], 1.8 min^− 1^ for *P. alba* [10], 5.28 min^− 1^ for *P. montana* [24], 0.66 min^− 1^ for *F. septica* [29], and 0.9 min^− 1^ for *C. equisetifolia* [29]. The *K*_*m*_ values of IspSs from *P. alba* [29], *F. septica* [29], *P. tremuloides* [12], *Salix discolor* [5], and *Quercus robur* [14] are 15.9, 3.4, 8, 8, and 0.53 mM respectively. Michaelis constants (*K*_*m*_) for IspS are usually very large compared to monoterpene or sesquiterpene synthases, which have apparent *K*_*m*_ values in the μM range [7]. The large *K*_*m*_ of isoprene synthases is evolutionarily advantageous by ensuring that carbon is first directed to essential physiological functions of the plant and only secondarily to isoprene production, because the main role of isoprene could be to alleviate abiotic stresses [7, 24]. Additionally, Sharkey proposed that isoprene serves to protect plant membranes against heat stress [37]. Eventually, for the biotechnological production of isoprene, the high *K*_*m*_ and low activity of IspSs should be engineered because the requirement of high substrate (DMAPP) concentrations could become an obstacle to achieving high rates of isoprene synthesis.

### Isoprene production by *E. coli* expressing MpIspS

**Fig. 6:**
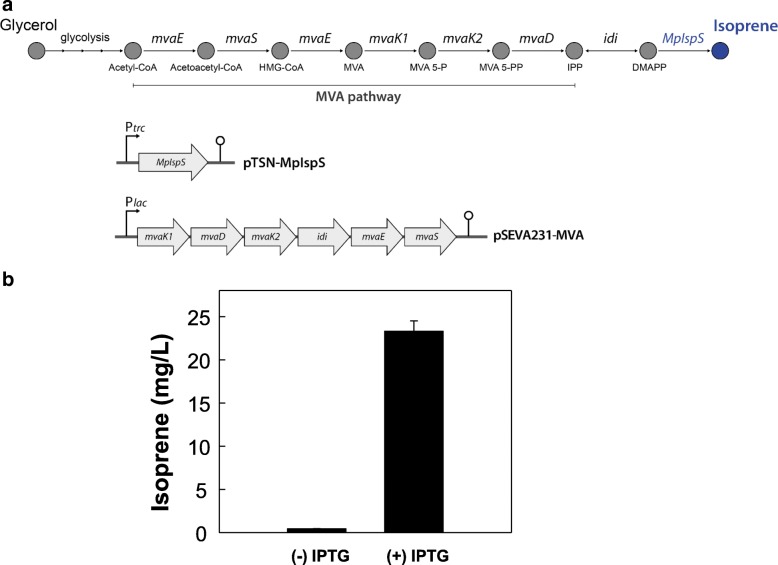
Isoprene production in *E. coli.* **a** Schematic representation of isoprene production pathway in an engineered *E. coli* harboring mevalonate (MVA) pathway genes. The isoprene production pathway from acetyl-CoA consists of seven enzymes: MvaE, Acetyl-CoA acetyltransferase/HMG-CoA reductase; MvaS, 3-hydroxy-3-methylglutaryl-CoA synthase; MvaK1, mevalonate kinase; MvaK2, phosphomevalonate kinase; MvaD, mevalonate 5-diphosphate decarboxylase; Idi, isopentenyl diphosphate isomerase; and MpIspS, isoprene synthase. All enzymes were expressed from IPTG-inducible promoters (P*trc* promoter for isoprene synthase, P*lac* promoter for mevalonate pathway). **b** Isoprene production at 30 °C by *E. coli* expressing MpIspS and MVA pathway genes with or without IPTG. Isoprene in the headspace was measured after 72 h incubation at 30 °C

Wild-type *E. coli* can produce isoprene by the introduction of plant-derived IspS because it possesses an endogenous methylerythritol phosphate (MEP) pathway that is capable of which producing DMAPP. However, the amount of isoprene that is produced is very low because of the limited availability of DMAPP. In other studies, the MVA pathway has been reconstructed with multiple genes derived from various bacteria and yeast, and co-expressed along with IspS to enhance isoprene production by supplying enough amount of DMAPP [15, 17, 19] (Fig. 6a). To determine the in vivo isoprene production by MpIspS from glycerol, an MpIspS expressing plasmid, pTSN-MpIspS was introduced into *E. coli* DH5α with the exogenous MVA pathway encoded by pSEVA231-MVA plasmid (Fig. 6a). The *E. coli* strain expressing MpIspS in the presence of IPTG (Isopropyl β- d − 1-thiogalactopyranoside) produced 23.3 mg/L at OD 4.6 ± 0.4 isoprene after 72-h cultivation whereas the strain expressing MpIspS without IPTG as a control produced 0.43 mg/mL at OD 6.1 ± 0.6 (Fig. 6b), which resulted from leaky expression of MpIspS under the control of *trc* promoter. As expected, isoprene was not produced in cells harboring the pTSN plasmid (no IspS) and the pSEVA231-MVA plasmid (MVA pathway) (Additional file 2: Figure S2). The optimal culture conditions for maximal isoprene production by MpIspS along with the MVA pathway should be further studied. Although IspS from *P. trichocarpa* has the highest isoprene productivity among the reported IspSs [19], MpIspS could potentially be an enzyme for isoprene synthesis in various microorganisms.

## Conclusions

In this work, we discovered a new IspS by sequence homology searches and identified the gene potentially encoding IspS *M. polymorpha.* The gene encoding the putative IspS from *M. polymorpha* was cloned, expressed in *E. coli*, purified, and characterized. Recombinant MpIspS demonstrated IspS activity with a preference for Mn^2+^ as a cofactor, and its optimum pH and temperature were 6.0 and 50 °C, respectively. The MpIspS was successfully used to produce isoprene from glycerol in *E. coli* expressing the heterologous MVA pathway genes. The MpIspS enzyme could be expressed in large amounts in *E. coli*, which led to increased enzymatic activity and resulted in high isoprene production in the *E. coli*. Based on our results, further protein engineering of MpIspS toward improved enzyme activity will pave the way for the high-level production of isoprene. Furthermore, engineered MpIspS in combination with the exogenous MVA pathway will be a platform for the enhanced production of isoprene in microorganisms. Overall, this study will provide a new IspS enzyme that is a useful key biocatalyst for isoprene production in engineered microbes.

## Methods

### Chemicals and materials

All chemical reagents used in this study were purchased from Sigma-Aldrich (St. Louis, MO, USA). Oligonucleotides and gene synthesis were provided by Macrogen (Seoul, Korea). Restriction endonucleases, polymerases, and DNA cloning kits were purchased from New England Biolabs (Ipswich, MA, USA). DNA preparation and manipulation techniques were carried out according to standard protocols for molecular biology. The kits of PCR product purification, gel extraction, and plasmid preparation were purchased from Promega (Madison, WI, USA). Profinia™ purification kits and all materials for SDS-PAGE were purchased from Bio-Rad (Hercules, CA, USA).

### Gene-mining of novel IspS

To mine the novel IspS from the transcriptome datasets of plants belonging the family Fabaceae, Salicaceae, and Myrtaceae, approximately 113 GB of unassembled reads from the transcriptome projects (PRJDB3212, PRJNA275266, PRJNA296824, PRJNA232948, PRJEB10287, PRJNA203514, and PRJEB11301) were downloaded from the Sequence Read Archive website of GenBank. BLASTX searches of individual reads (E-value ≤1e^− 10^) were performed against an in-house database consisting of IspSs and other terpene synthases retrieved from the GenBank database [38]. Reads assigned to IspS and other terpene synthases were extracted and sorted into sub-datasets of IspS and the other group. Reads in the IspS sub-datasets from each organism were assembled into six contigs using a sequence assembler. Open reading frames from each scaffold were identified using Prodigal v2.6.1 [39]. The predicted open reading frames were BLAST-searched against the UniProt databases [40]. Candidate isoprene synthase sequences were aligned using MUSCLE algorithm in the MEGA6 package program v.6.06 [41].

### Gene synthesis and cloning of MpIspS

The *MpIspS* (*IspS* from *Metrosideros polymorpha*) gene identified from gene mining was codon-optimized for *E. coli* using the by IDT Codon Optimization Tool (Integrated DNA Technologies Inc., Coralville, IA, USA; http://sg.idtdna.com/CodonOpt) and synthesized by Macrogen co. (Seoul, South Korea) (Additional file 3: Table S1). *E. coli* C2566 (New England Biolabs) and the pET-28a(+) plasmid (Novagen, Merck KGaA, Darmstadt, Germany) were used as host cells and expression vectors, respectively. The gene encoding MpIspS was amplified by PCR using the synthetic DNA as the template*.* The MpIspS coding region was cloned between the T7 promoter and terminator in the pET-28a(+) plasmid containing an N-terminal His_6_ tag. Forward (5′-CGGCAGCCATATGTGTAGTG-3′) and reverse (5′-GGTGGTGCTCGAGTTAACGCGG-3′) primers were designed for the introduction of the *Nde*I and *Xho*I restriction sites (underlined), respectively. The PCR product was subcloned into the pET-28a(+) plasmid digested with the same restriction enzymes and then transformed into *E. coli* C2566. For isoprene production in *E. coli*, we constructed an MpIspS-expressing plasmid (pTSN-MpIspS). Forward (5′-ACACAGGAGGTTAAACCATGTGTAGTGCTTCCACACAAGT-3′) and reverse (5′-CATGCCTGCAGGTCGACTCTAGATTAACGC-3′) primers were designed and the MpIspS coding region was amplified by PCR using the synthetic DNA as the template. Vector backbone was prepared after digestion of the pTSN plasmid [42] with *Nco*I and *Xba*I, and then the PCR product was ligated into the digested pTSN plasmid using the Gibson Assembly Master Mix (New England Biolabs). For MVA pathway expression, we constructed the pSEVA231-MVA plasmid. The first fragment containing the *lacI* gene was amplified using forward (5′-TCACACAGGACGAAGCGGCATGCATTTACG-3′) and reverse (5′-GCGTTCGAACGGCAGAATTGCAGCTCATTTCAGAATATTT-3′) primers from the pSNA-MrBBS plasmid containing MVA pathway genes [42], the second fragment containing MVA pathway genes was amplified using forward (5′-CAATTCTGCCGTTCGAACGCTAATCTAGAGCGCAACGCAA-3′) and reverse (5′-CAGTCACGACAAGAGTTTGTAGAAACGCAA-3′) primers from the pSNA-MrBBS plasmid as template, and the third fragment as vector backbone was amplified using forward (5′-ACAAACTCTTGTCGTGACTGGGAAAACCCT-3′) and reverse (5′- TGCCGCTTCGTCCTGTGTGAAATTGTTATC-3′) primers from the pSEVA231 plasmid [43]. The three fragments were assembled using the Gibson Assembly Master Mix (New England Biolabs). The correctness of the constructed plasmid was confirmed by Sanger sequencing (Macrogen).

### MpIspS purification

The MpIspS expressing cells were harvested from culture broth and disrupted on ice using ultrasonication (Thermo Fisher Scientific, Waltham, MA, USA) in buffer A (50 mM sodium monophosphate, 300 mM NaCl, 10 mM imidazole, and 0.1 mM phenylmethylsulfonyl fluoride as a protease inhibitor). Unbroken cells and cell debris were removed by centrifugation at 14,000 rpm for 10 min at 4 °C, and the supernatants were filtered through a 0.45 μm filter and applied to an IMAC column (Bio-Rad) equilibrated with buffer A. Supernatants collected from lysates were loaded into the Profinia™ Purification System (Bio-Rad). Supernatants were loaded onto a 1-mL IMAC cartridge and washed twice with 5 and 10 mM imidazole buffer A. Proteins were eluted with 250 mM imidazole in buffer A. Imidazole and other salts were removed and changed with 50 mM MOPS buffer (pH 6.0) using a desalting cartridge. The resulting solution was used as the purified MpIspS enzyme. The protein concentration was quantified by the standard Bradford method [44]. The purified proteins were confirmed by SDS-PAGE.

### Molecular mass determination of MpIspS

The subunit molecular mass of MpIspS was examined by SDS-PAGE under denaturing conditions, using the proteins of a pre-stained ladder (Bio-Rad) as reference proteins. All protein bands were stained with Coomassie Blue for visualization. The native molecular mass of the enzyme was determined by gel-filtration chromatography on a Superose 12 10/300 GL column (GE Healthcare, Buckinghamshire, UK). The purified enzyme was applied to the column and eluted with 25 mM Tris-HCl (pH 7.4) buffer containing 200 mM NaCl at a flow rate of 1 mL/min. The column was calibrated with apoferritin (443 kDa), β-amylase (200 kDa), alcohol dehydrogenase (150 kDa), albumin (13.7 kDa), and carbonic anhydrase (29 kDa) as reference proteins (Sigma-Aldrich), and the native molecular mass of the enzyme was calculated by comparing with the migration length of reference proteins.

### Effects of metal ions, pH, and temperature

To evaluate the effect of metal ions on enzyme activity, an enzyme assay was conducted after the treatment with 1 mM ethylenediaminetetraacetic acid at 4 °C for 1 h or after addition a 1 mM concentration of each metal ion (Mn^2+^, Zn^2+^, Cu^2+^, Ni^2+^, Co^2+^, Mg^2+^, Ca^2+^, or Fe^2+^). The reactions were performed in 50 mM MOPS buffer (pH 6.0) containing each metal ion at 50 °C. To examine the effect of pH on the activity of MpIspS, the pH was varied between 4.5 and 7.5 using 50 mM sodium citrate (pH 4.5–6.0) and 50 mM MOPS buffer (3-(N-morpholino)propanesulfonic acid; pH 6–7.5) containing 5 mM Mn^2+^ or 1 mM Mg^2+^. To investigate the effect of temperature on MpIspS enzyme activity, the temperature was varied from 30 to 60 °C. One unit (U) of MpIspS activity was defined as the amount of enzyme required to produce 1 μM of isoprene per min at 50 °C and pH 6.0.

### Analytical method

To measure isoprene concentration as described previously [30], 50 μL headspace of the sealed serum bottle used for the enzyme reaction or the cultivation of the engineered *E. coli* was directly injected into the GC system equipped with a flame ionization detector (FID) and an HP-5 column (30 m × 0.320 mm × 0.25 μm) at a flow rate of 1 mL/min. The starting temperature of the oven was maintained at 40 °C for 3 min, then was increased by 10 °C/min to 100 °C, held at 100 °C for 3 min, increased at the rate of 30 °C/min to 200 °C, and then again held at 200 °C for 1 min. Commercial isoprene (Sigma-Aldrich) was used as an external standard for the quantification of isoprene. The retention time (R.T.) of the standard isoprene was 2.8 min.

### Kinetic parameters

Various concentrations of DMAPP (0–10 mM) were used to determine the kinetic parameters of the MpIspS enzyme. The reaction was conducted in 50 mM MOPS buffer (pH 6.0) containing 5 mM Mn^2+^ at 50 °C for 10 min. The amounts of isoprene in the headspace were detected by GC-FID. The enzyme kinetic parameters, *K*_*m*_ and *k*_cat_ values for substrates, were determined by fitting the data to the Michaelis–Menten equation.

### Bioisoprene production by *E. coli* expression with MpIspS

For isoprene production in *E. coli*, pTSN-MpIspS, and pSEVA231-MVA were co-transformed into *E. coli* DH5α. Transformants were selected on LB (10 g/L tryptone, 5 g/L yeast extract, and 10 g/L NaCl) agar plates containing 100 μg/mL ampicillin and 25 μg/mL kanamycin and incubated at 30 °C for 16 h. To prepare the seed culture, a single colony was cultivated in LB media containing 100 μg/mL ampicillin and 25 μg/mL kanamycin at 30 °C for 16 h. Culture for isoprene production was conducted in a sealed bottle containing 500 μL TB medium (24 g/L yeast extract, 12 g/L tryptone, 9.2 g/L K_2_HPO_4_, and 2.2 g/L KH_2_PO_4_) containing 0.025 mM IPTG, 100 μg/mL ampicillin, 25 μg/mL kanamycin, and 3.5% (*w*/*v*) glycerol as the main carbon source at 30 °C for 3 days. After cultivation, isoprene in the headspace was analyzed by GC-FID.

## Additional files

Additional file 1: **Figure S1.** Effects of pH and temperature on activity of MpIspS. A. pH: The reactions were performed in 50 mM sodium citrate (circle) or 50 mM MOPS buffer (square) containing 50 μM DMAPP and 5 mM Mn^2+^ (closed symbol) or 1 mM Mg^2+^ (open symbol) at 55 °C for 10 min. B. Temperature: Reactions were performed in 50 mM MOPS buffer (pH 6.0) containing 50 μM DMAPP and 5 mM Mn^2+^ for 10 min. *Relative activity of 100% was 17.5271 U/mg. (PPT 141 kb)

Additional file 2: **Figure S2.** GC-MS analysis of isoprene from the head space in culture for MPIspS3 and MVA pathway harboring *E. coli*. Isoprene, MPIspS3 product, and empty vector represent commercial isoprene as standard, product from MPIspS3 and MVA pathway harboring *E. coli*, and product from pET28a(+) and MVA pathway harboring *E. coli*, respectively. (PPT 186 kb)

Additional file 3: **Table S1.** Gene sequences of IspS from *Metrosideros polymorpha*. (PPT 140 kb)

## Abbreviations

DMAPP: Dimethylallyl diphosphate
IPTG: Isopropyl β- d − 1-thiogalactopyranoside
IspS: Isoprene synthase
MEP: Methylerythritol phosphate
MpIspS: Isoprene synthase from *Metrosideros polymorpha*
MVA: Mevalonate
