# Supplementary material for: Molecular and biochemical characterization of a novel isoprene synthase from Metrosideros polymorpha
Source: BMC Plant Biol. 2018 Jun 15;18:118. doi: 10.1186/s12870-018-1315-4 (PMC6003189; doi:10.1186/s12870-018-1315-4)
Supplement: Supplementary file 1 — Figure S1. Effects of pH and temperature on activity of MpIspS. A. pH: The reactions were performed in 50 mM sodium citrate (circle) or 50 mM MOPS buffer (square) containing 50 μM DMAPP and 5 mM Mn2+ (closed symbol) or 1 mM Mg2+ (open symbol) at 55 °C for 10 min. B. Temperature: Reactions were performed in 50 mM MOPS buffer (pH 6.0) containing 50 μM DMAPP and 5 mM Mn2+ for 10 min. *Relative activity of 100% was 17.5271 U/mg. (PPT 141 kb) [file 12870_2018_1315_MOESM1_ESM.ppt]

## Slide 1
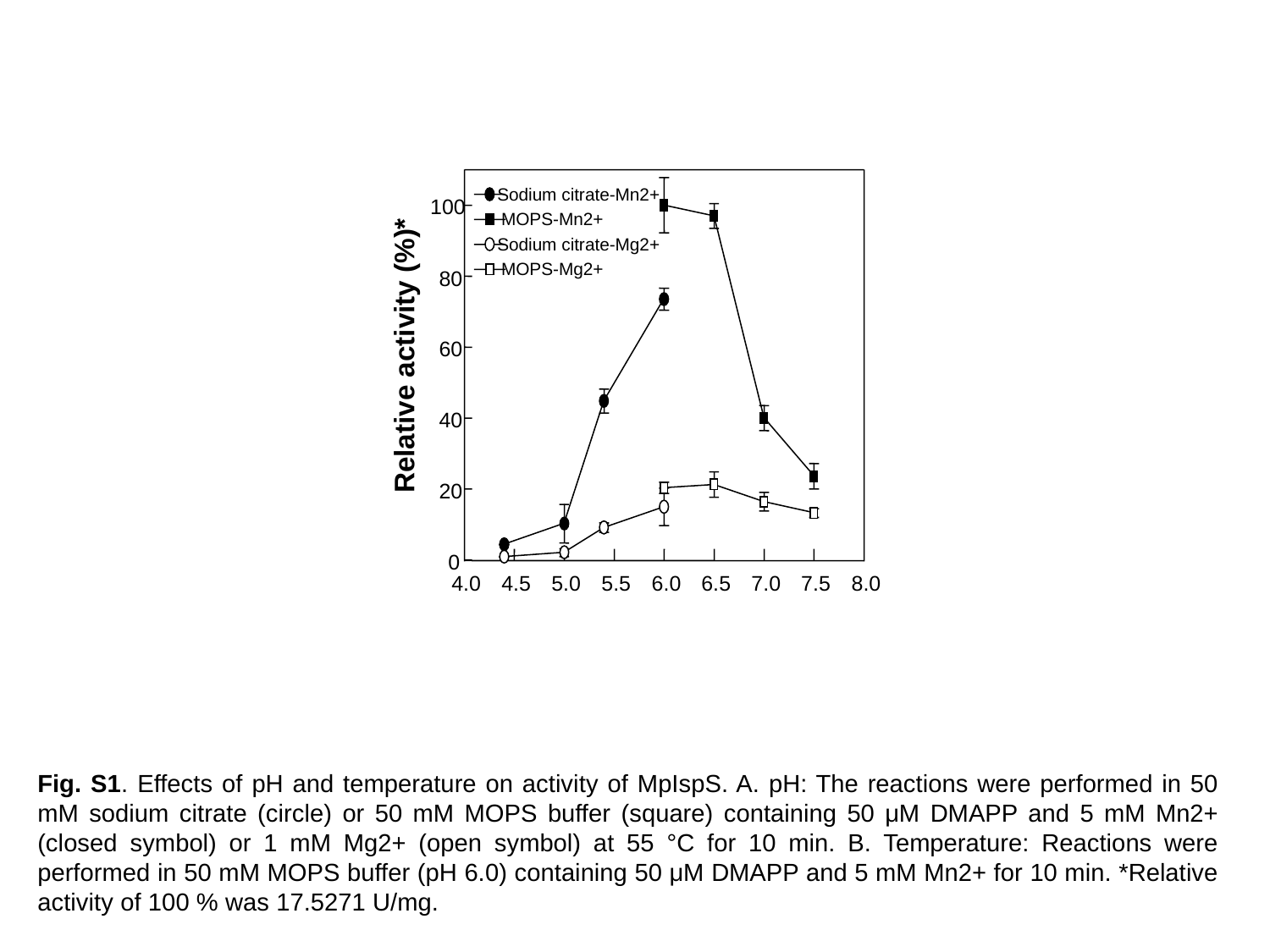

Sodium citrate-Mn2+
MOPS-Mn2+
Sodium citrate-Mg2+
MOPS-Mg2+
100
80
60
Relative activity (%)*
40
20
0
4.0
4.5
5.0
5.5
6.0
6.5
7.0
7.5
8.0
Fig. S1. Effects of pH and temperature on activity of MpIspS. A. pH: The reactions were performed in 50 mM sodium citrate (circle) or 50 mM MOPS buffer (square) containing 50 μM DMAPP and 5 mM Mn2+ (closed symbol) or 1 mM Mg2+ (open symbol) at 55 °C for 10 min. B. Temperature: Reactions were performed in 50 mM MOPS buffer (pH 6.0) containing 50 μM DMAPP and 5 mM Mn2+ for 10 min. *Relative activity of 100 % was 17.5271 U/mg.
