# Supplementary material for: Molecular and biochemical characterization of a novel isoprene synthase from Metrosideros polymorpha
Source: BMC Plant Biol. 2018 Jun 15;18:118. doi: 10.1186/s12870-018-1315-4 (PMC6003189; doi:10.1186/s12870-018-1315-4)
Supplement: Supplementary file 2 — Figure S2. GC-MS analysis of isoprene from the head space in culture for MPIspS3 and MVA pathway harboring E. coli. Isoprene, MPIspS3 product, and empty vector represent commercial isoprene as standard, product from MPIspS3 and MVA pathway harboring E. coli, and product from pET28a(+) and MVA pathway harboring E. coli, respectively. (PPT 186 kb) [file 12870_2018_1315_MOESM2_ESM.ppt]

## Slide 1
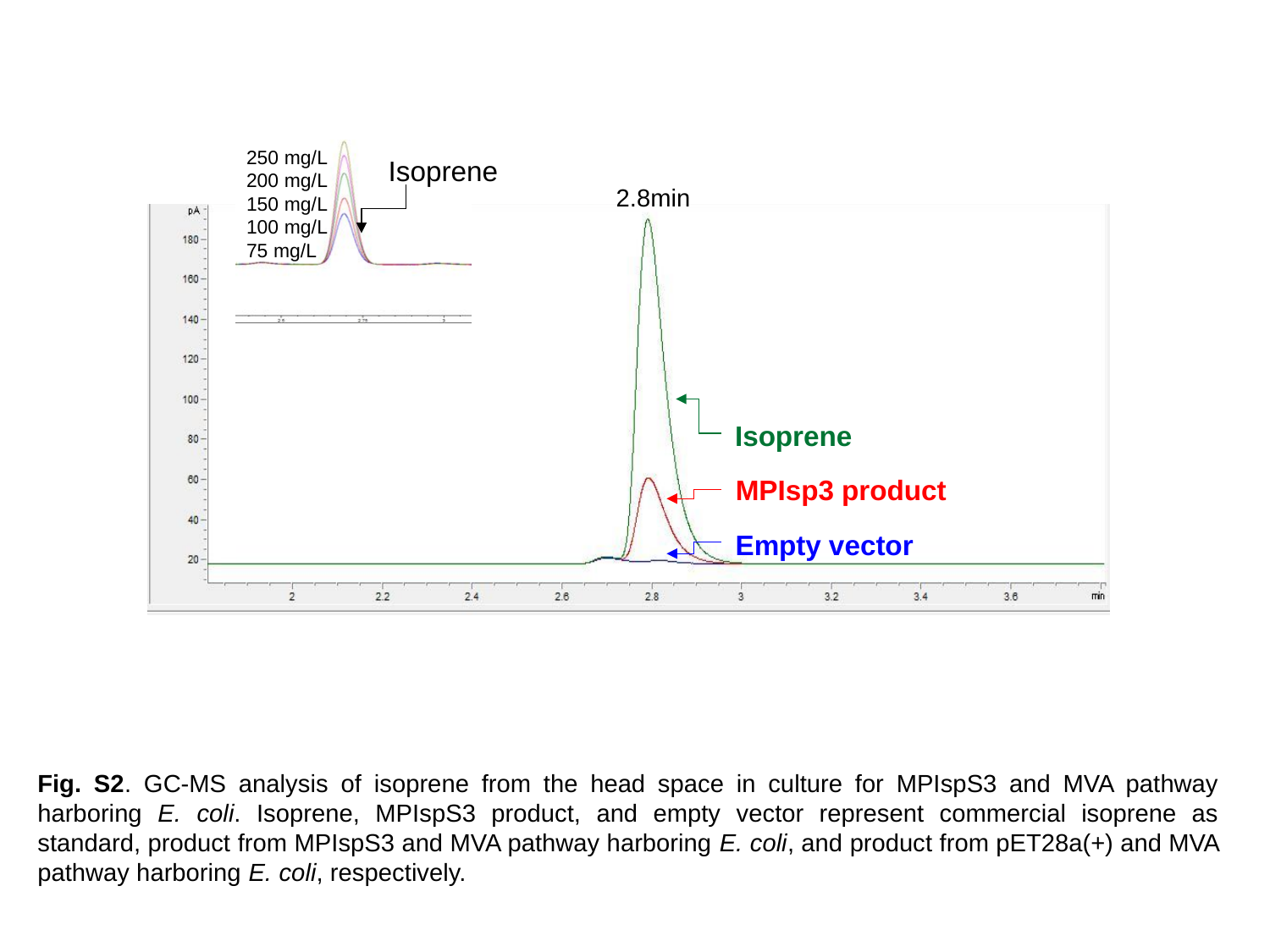

250 mg/L
200 mg/L
150 mg/L
100 mg/L
75 mg/L
Isoprene
2.8min
Isoprene
MPIsp3 product
Empty vector
Fig. S2. GC-MS analysis of isoprene from the head space in culture for MPIspS3 and MVA pathway harboring E. coli. Isoprene, MPIspS3 product, and empty vector represent commercial isoprene as standard, product from MPIspS3 and MVA pathway harboring E. coli, and product from pET28a(+) and MVA pathway harboring E. coli, respectively.
