# Supplementary material for: Molecular and biochemical characterization of a novel isoprene synthase from Metrosideros polymorpha
Source: BMC Plant Biol. 2018 Jun 15;18:118. doi: 10.1186/s12870-018-1315-4 (PMC6003189; doi:10.1186/s12870-018-1315-4)
Supplement: Supplementary file 3 — Table S1. Gene sequences of IspS from Metrosideros polymorpha. (PPT 140 kb) [file 12870_2018_1315_MOESM3_ESM.ppt]

## Slide 1
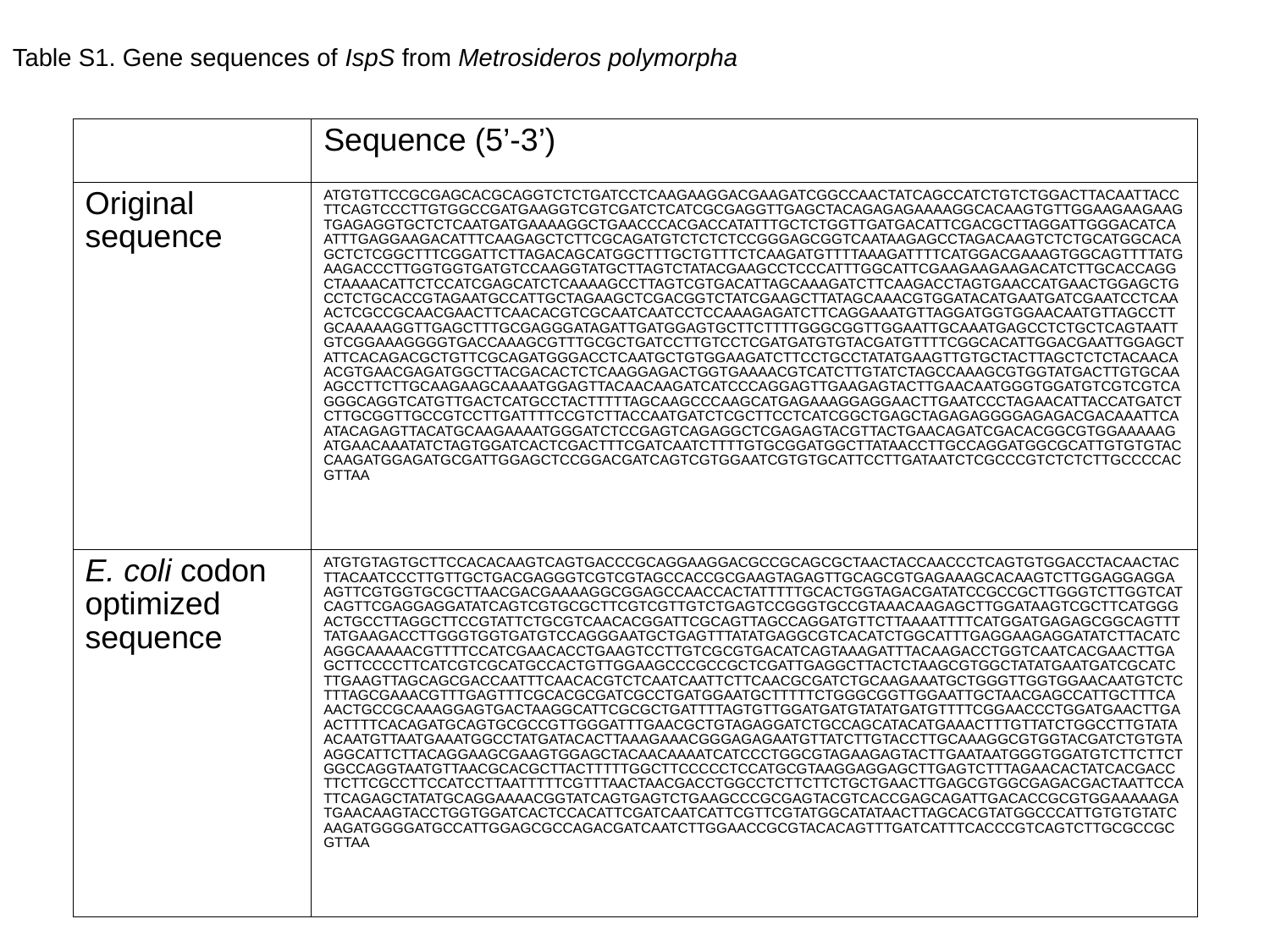

Table S1. Gene sequences of IspS from Metrosideros polymorpha
| | Sequence (5’-3’) |
| --- | --- |
| Original sequence | ATGTGTTCCGCGAGCACGCAGGTCTCTGATCCTCAAGAAGGACGAAGATCGGCCAACTATCAGCCATCTGTCTGGACTTACAATTACCTTCAGTCCCTTGTGGCCGATGAAGGTCGTCGATCTCATCGCGAGGTTGAGCTACAGAGAGAAAAGGCACAAGTGTTGGAAGAAGAAGTGAGAGGTGCTCTCAATGATGAAAAGGCTGAACCCACGACCATATTTGCTCTGGTTGATGACATTCGACGCTTAGGATTGGGACATCAATTTGAGGAAGACATTTCAAGAGCTCTTCGCAGATGTCTCTCTCCGGGAGCGGTCAATAAGAGCCTAGACAAGTCTCTGCATGGCACAGCTCTCGGCTTTCGGATTCTTAGACAGCATGGCTTTGCTGTTTCTCAAGATGTTTTAAAGATTTTCATGGACGAAAGTGGCAGTTTTATGAAGACCCTTGGTGGTGATGTCCAAGGTATGCTTAGTCTATACGAAGCCTCCCATTTGGCATTCGAAGAAGAAGACATCTTGCACCAGGCTAAAACATTCTCCATCGAGCATCTCAAAAGCCTTAGTCGTGACATTAGCAAAGATCTTCAAGACCTAGTGAACCATGAACTGGAGCTGCCTCTGCACCGTAGAATGCCATTGCTAGAAGCTCGACGGTCTATCGAAGCTTATAGCAAACGTGGATACATGAATGATCGAATCCTCAAACTCGCCGCAACGAACTTCAACACGTCGCAATCAATCCTCCAAAGAGATCTTCAGGAAATGTTAGGATGGTGGAACAATGTTAGCCTTGCAAAAAGGTTGAGCTTTGCGAGGGATAGATTGATGGAGTGCTTCTTTTGGGCGGTTGGAATTGCAAATGAGCCTCTGCTCAGTAATTGTCGGAAAGGGGTGACCAAAGCGTTTGCGCTGATCCTTGTCCTCGATGATGTGTACGATGTTTTCGGCACATTGGACGAATTGGAGCTATTCACAGACGCTGTTCGCAGATGGGACCTCAATGCTGTGGAAGATCTTCCTGCCTATATGAAGTTGTGCTACTTAGCTCTCTACAACAACGTGAACGAGATGGCTTACGACACTCTCAAGGAGACTGGTGAAAACGTCATCTTGTATCTAGCCAAAGCGTGGTATGACTTGTGCAAAGCCTTCTTGCAAGAAGCAAAATGGAGTTACAACAAGATCATCCCAGGAGTTGAAGAGTACTTGAACAATGGGTGGATGTCGTCGTCAGGGCAGGTCATGTTGACTCATGCCTACTTTTTAGCAAGCCCAAGCATGAGAAAGGAGGAACTTGAATCCCTAGAACATTACCATGATCTCTTGCGGTTGCCGTCCTTGATTTTCCGTCTTACCAATGATCTCGCTTCCTCATCGGCTGAGCTAGAGAGGGGAGAGACGACAAATTCAATACAGAGTTACATGCAAGAAAATGGGATCTCCGAGTCAGAGGCTCGAGAGTACGTTACTGAACAGATCGACACGGCGTGGAAAAAGATGAACAAATATCTAGTGGATCACTCGACTTTCGATCAATCTTTTGTGCGGATGGCTTATAACCTTGCCAGGATGGCGCATTGTGTGTACCAAGATGGAGATGCGATTGGAGCTCCGGACGATCAGTCGTGGAATCGTGTGCATTCCTTGATAATCTCGCCCGTCTCTCTTGCCCCACGTTAA |
| E. coli codon optimized sequence | ATGTGTAGTGCTTCCACACAAGTCAGTGACCCGCAGGAAGGACGCCGCAGCGCTAACTACCAACCCTCAGTGTGGACCTACAACTACTTACAATCCCTTGTTGCTGACGAGGGTCGTCGTAGCCACCGCGAAGTAGAGTTGCAGCGTGAGAAAGCACAAGTCTTGGAGGAGGAAGTTCGTGGTGCGCTTAACGACGAAAAGGCGGAGCCAACCACTATTTTTGCACTGGTAGACGATATCCGCCGCTTGGGTCTTGGTCATCAGTTCGAGGAGGATATCAGTCGTGCGCTTCGTCGTTGTCTGAGTCCGGGTGCCGTAAACAAGAGCTTGGATAAGTCGCTTCATGGGACTGCCTTAGGCTTCCGTATTCTGCGTCAACACGGATTCGCAGTTAGCCAGGATGTTCTTAAAATTTTCATGGATGAGAGCGGCAGTTTTATGAAGACCTTGGGTGGTGATGTCCAGGGAATGCTGAGTTTATATGAGGCGTCACATCTGGCATTTGAGGAAGAGGATATCTTACATCAGGCAAAAACGTTTTCCATCGAACACCTGAAGTCCTTGTCGCGTGACATCAGTAAAGATTTACAAGACCTGGTCAATCACGAACTTGAGCTTCCCCTTCATCGTCGCATGCCACTGTTGGAAGCCCGCCGCTCGATTGAGGCTTACTCTAAGCGTGGCTATATGAATGATCGCATCTTGAAGTTAGCAGCGACCAATTTCAACACGTCTCAATCAATTCTTCAACGCGATCTGCAAGAAATGCTGGGTTGGTGGAACAATGTCTCTTTAGCGAAACGTTTGAGTTTCGCACGCGATCGCCTGATGGAATGCTTTTTCTGGGCGGTTGGAATTGCTAACGAGCCATTGCTTTCAAACTGCCGCAAAGGAGTGACTAAGGCATTCGCGCTGATTTTAGTGTTGGATGATGTATATGATGTTTTCGGAACCCTGGATGAACTTGAACTTTTCACAGATGCAGTGCGCCGTTGGGATTTGAACGCTGTAGAGGATCTGCCAGCATACATGAAACTTTGTTATCTGGCCTTGTATAACAATGTTAATGAAATGGCCTATGATACACTTAAAGAAACGGGAGAGAATGTTATCTTGTACCTTGCAAAGGCGTGGTACGATCTGTGTAAGGCATTCTTACAGGAAGCGAAGTGGAGCTACAACAAAATCATCCCTGGCGTAGAAGAGTACTTGAATAATGGGTGGATGTCTTCTTCTGGCCAGGTAATGTTAACGCACGCTTACTTTTTGGCTTCCCCCTCCATGCGTAAGGAGGAGCTTGAGTCTTTAGAACACTATCACGACCTTCTTCGCCTTCCATCCTTAATTTTTCGTTTAACTAACGACCTGGCCTCTTCTTCTGCTGAACTTGAGCGTGGCGAGACGACTAATTCCATTCAGAGCTATATGCAGGAAAACGGTATCAGTGAGTCTGAAGCCCGCGAGTACGTCACCGAGCAGATTGACACCGCGTGGAAAAAGATGAACAAGTACCTGGTGGATCACTCCACATTCGATCAATCATTCGTTCGTATGGCATATAACTTAGCACGTATGGCCCATTGTGTGTATCAAGATGGGGATGCCATTGGAGCGCCAGACGATCAATCTTGGAACCGCGTACACAGTTTGATCATTTCACCCGTCAGTCTTGCGCCGCGTTAA |
